# Achieving water-floatable photocatalyst on recycled bamboo chopsticks

**DOI:** 10.1038/s41598-024-60272-7

**Published:** 2024-04-25

**Authors:** Sujun Guan, Lijun Wang, Liang Hao, Hiroyuki Yoshida, Takaomi Itoi, Yun Lu, Chiaki Terashima, Akira Fujishima

**Affiliations:** 1 Research Center for Space System Innovation, Tokyo University of Science, Chiba, 2788510 Japan; 2 School of Intelligent Manufacturing, Chengdu Technological University, Chengdu, 610031 China; 3 College of Mechanical Engineering, Tianjin University of Science and Technology, Tianjin, 300222 China; 4 Chiba Industrial Technology Research Institute, Chiba, 2630016 Japan; 5 Graduate School and Faculty of Engineering, Chiba University, Chiba, 2638522 Japan; 6 Department of Pure and Applied Chemistry, Tokyo University of Science, Chiba, 2788510 Japan; 7 Shanghai Institute of Photocatalysis Industrial Technology, University of Shanghai for Science and Technology, Shanghai, 200093 China

**Keywords:** Waste recycling, Floatable photocatalyst, TiO_2_, TiC, Heterojunction, Visible light response, Photocatalysis, Pollution remediation

## Abstract

Disposable bamboo chopsticks (DBCs) are difficult to recycle, which inevitably cause secondary pollution. Based on energy and environmental issues, we propose a facile strategy to fabricate floatable photocatalyst (fPC) coated onto DBCs, which can be flexibly used in water purification. The photocatalyst of titania and titanium carbide on bamboo (TiO_2_/TiC@b) was successfully constructed from TiC-Ti powders and DBCs using a coating technique followed heat treatment in carbon powder, and the fPC exhibited excellent photocatalytic activity under visible light irradation. The analysis results indicate that rutile TiO_2_ forms on TiC during heat treatment, achieving a low-density material with an average value of approximately 0.5233 g/cm^3^. The coatings of TiO_2_/TiC on the bamboo are firm and uniform, with a particle size of about 20–50 nm. XPS results show that a large amount of oxygen vacancies is generated, due to the reaction atmosphere of more carbon and less oxygen, further favoring to narrowing the band gap of TiO_2_. Furthermore, TiO_2_ formed on residual TiC would induce the formation of a heterojunction, which effectively inhibits the photogenerated electron–hole recombination via the charge transfer effect. Notably, the degradation of dye Rhodamine B (Rh.B) is 62.4% within 3 h, while a previous adsorption of 36.0% for 1 h. The excellent photocatalytic performance of TiO_2_/TiC@b can be attributed to the enhanced reaction at the water/air interface due to the reduced light loss in water, improved visible-light response, increased accessible area and charge transfer effect. Our findings show that the proposed strategy achieves a simple, low-cost, and mass-producible method to fabricate fPC onto the used DBCs, which is expected to applied in multiple fields, especially in waste recycling and water treatment.

## Introduction

The increasing amount of municipal waste is a global crisis caused by the huge consumption of resources due to poor operations and limited capacity, thereby affecting the ecosystem and the quality of human life^1–3^. Especially areas influenced by Asian food culture, the use of chopsticks inevitably causes a great waste pollution, and disposable chopsticks bear the brunt. Unfortunately, there are few recycling options and policies for disposable chopsticks, which further makes disposable chopsticks difficult to recycle^4,5^. In addition, DBCs are the most popular and widely used in Asia countries, and continue to expand further globally. As disposable tableware, DBCs are convenient, cheap, and hygienic, but a pair of chopsticks can only emply for one meal, causing a high environmental burden. Therefore, disposing of DBCs is both urgent and challenging, especially in Asian countries^6,7^. Considerable efforts have been made to study and convert DBC waste using various techniques, such as thermal-chemical decomposition, torrefaction with coal and hydrothermal treatment^8–12^. Bamboo charcoal, which can be fabricated through pyrolysis or carbonization processes, usually exhibits high conductivity, large specific surface area and adsorption performance. Hameed et al.^8^ and Liu et al.^9^ revealed that the activated bamboo-based carbon exhibited fast adsorption of methylene blue (MB) dye. Wei et al.^10^ and Ji et al.^11^ reported that high adsorption capacity of bamboo biochar could be served as a CO_2_ capture. On the background of global energy crisis, converting or preparing other new energy sources is more efficient than recycling resources, and has received high attention. Jiang et al. proposed a scalable and smart approach to evolve DBCs waste into useful carbon fibers, which could be applied for Li-ion batteries, biosensors, etc.^12^. Recently, Li et al. demonstrated that a smart and highly efficient catalytic microreactor, with encaplsulating Pd-TiO_2_ into bamboo microchannels, using for the continuous-flow hydrogenation reactions^13^.

Furthermore, environmental pollution, especially water pollution, is also very serious. Water plays a key role for human beings, while many reports show that half of the current world population could live in water-scarce areas, which are of course attributed to multiple reasons, but the first and foremost reason is the vast amount of untreated wastewater^14–16^. It is well known that when photocatalyst is exposed to light, the oxidation and reduction reactions occur on the surface of photocatalyst, which can be used in variety of fields, such as environmental purification, self-cleaning surfaces, hydrogen energy production via water splitting^17–20^. In photocatalyst materials, TiO_2_ has been widely used to solve environmental problems, owing to its suitable UV and visible light response, excellent chemical stability, resource-rich and satisfactory photocatalytic activity^19–22^. To absorb light energy more efficiently in practical application, the floatable photocatalyst is more efficient and shows many advantages, such as the enhanced reaction at the interface water/air, the reduced light loss in water^23–27^. Recently, Huang et al. demonstrated ultralight triphase photocatalytic system and novel aerogel with oxygen deliver channels and triphase interfaces, achieving efficient water purification, which was of constructive significance for the sustainable environmental development^26^.

In our previous works, highly firm photocatalyst coatings formed on ceramic balls were fabricated by mechanical coating technique and various heat treatment processes, and the photocatalyst coatings had been systematically investigated and exhibited satisfactory photocatalytic activity^28–30^. Based on DBCs recycling and environmental purification, herein, we propose a smart and scalable strategy to make waste DBCs to highly firm and efficient fPC. The fPC of TiO_2_/TiC coatings formed on bamboo (TiO_2_/TiC@b) are fabricated by the proposed coating method followed heat treatment in carbon powder, and the fPC shows excellent photocatalytic activity, especially under visible light irradation. TiO_2_/TiC@b possesses some unique advantages, such as light-weight, heterojunction, and recycling waste to photocatalyst. Furthermore, the mechanism of the photocatalytic reaction and degradation pathway of dye were investigated and discussed.

## Experimental

### fPC of TiO_*2*_/TiC@b

Ti powder (average diameter: 30 μm, Osaka Titanium technologies, ≥ 99.1%) and TiC nanopowder (average diameter: 40 nm, Sky Spring Nanomaterials, ≥ 99%) were used as the coating materials. The DBCs were collected from campus restaurant, and cut into bamboo blocks with length of 5–8 mm for the next use after a wash-dry process. Alumina (Al_2_O_3_) balls (average diameter: 1 mm, Nikkato, ≥ 98.5%) and the cut DBCs were used as coating substrates. Carbon powder (average diameter: 110 μm, Graphite Ito Co., ≥ 99.9%) was employed during heat treatment in carbon powder.

Firstly, the coating materials of TiC powder (20 g) and Ti powder (80 g), and the coating substrates of bamboo blocks (20 g) and Al_2_O_3_ balls (380 g) were charged into an Al_2_O_3_ bowl (dimensions: *Φ* 100 mm × 75 mm, 500 mL). TiC-Ti composite coatings were coated on bamboo blocks by the proposed coating method with a 400-rpm rotation speed for 1.5 h, marked as "20TiC@b". More detailed coating method can be found in our previous work^28–30^. Meanwhile, Ti coatings were also coated on bamboo blocks under the same coating conditions, marked as "Ti@b". Subsequently, the prepared samples were cleaned in water with an ultrasonic cleaner (frequency: 40 kHz, As one US-2R) for 10 min to remove the possible powders that may easily fall off. During the following heat treatment, the 20TiC@b and Ti@b samples were embedded in carbon powder respectively, then performed by electric furnace (HPM-1G, As one) at 600 °C for 0.5 h. The treated samples were kept in the furnace until the temperature dropped to approximately 25 °C, and marked as "20TiC-cHT" and "Ti-cHT", respectively.

### Characterization

The change in the formed rutile TiO_2_ on TiC coatings and their crystal structures were investigated by X-ray diffraction (XRD, Rigaku Ultima IV), recording from 25 to 63 degrees within a 0.02 deg/s step. Surface morphology was examined using scanning electron microscopy (SEM, Hitachi SU8030), equipped with energy-dispersive X-ray spectroscopy (EDS), and the cross-section was observed using an optical microscope (OM, Keyence XG X-1000). An ultraviolet–visible spectrophotometer (UV–vis, V-670, Jasco) was used to measure the ultraviolet–visible absorption spectra from 330 to 700 nm. X-ray photoelectron spectroscopy (XPS, PHI Quantes) measurements were used to investigate the change in chemical composition on the surface.

### Photocatalytic activity evaluation

Rhodamine B (Rh.B) was chosen as the photodecomposition dye in the photocatalytic activity evaluation the fPC under visible light irradiation at approximately 25 °C. The samples were spread uniformly on the bottom of the cell, then slowly poured the Rh.B solution (*C*_*0*_: 10 mg/L, 30 mL) into the cell. To reach an adsorption–desorption equilibrium of RhB molecules, the cells containing the RhB solution and the samples were kept in dark for 60 min. The photocatalytic activity test was then carried out under a light source with an irradiance of 5000 lx, using two 20 W fluorescent lamps (FL20SSW/18, NEC), equipped a cut-off filter (L42, Hoya). The decomposition of the RhB solution was monitored by a digital colorimeter (mini photo 10; Sanshin Kogyo) every 30 min. Moreover, the adsorbed amount onto the samples at equilibrium (Q, mg/g) was calculated by:

$${\text{Q}} = ({\text{C}}_{0} - {\text{C}}_{{\text{e}}} ) \times {\text{V}}/{\text{m}}$$

where Q is the quantity of Rh.B adsorbed by mass of fPC (mg/L), C_0_ is the initial concentration of Rh.B (mg/L), C_e_ is the concentration at the equilibrium (mg/L), V is the solution volume (L), and m is the mass of the fPC (g/L)^31,32^. The photodegradation efficiency (%) was calculated from:

$${\text{R }}\left( \% \right) \, = \, \left( {{\text{C}}_{{\text{e}}} - {\text{C}}_{{\text{t}}} } \right)/{\text{C}}_{{\text{e}}} \times { 1}00\% ,$$

where C_t_ is concentration at the test-time (mg/L)^32,33^. To evaluate the photocatalytic stability, the cycle tests were performed. Each cycle test was restarted with a fresh Rh.B solution^30^.

### Ethical standards

This study does not involve any human participants and/or animals.

## Results and discussion

### Appearance and crystal structure

**Figure 1:**
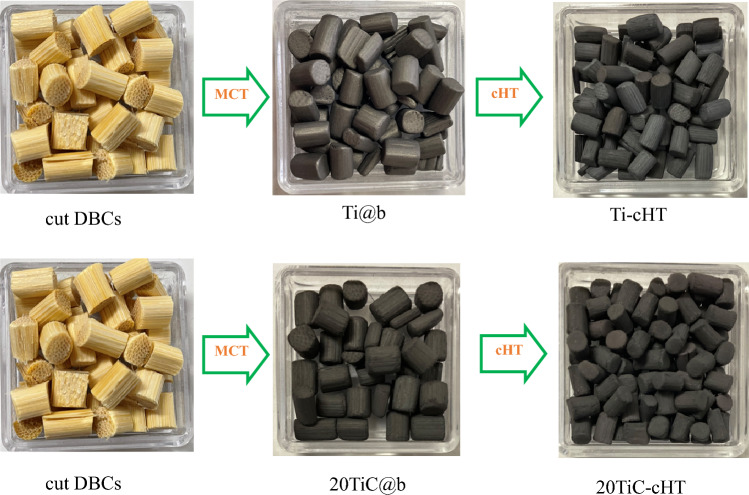
Schematic illustrating samples and their dye decomposition. UV: ultraviolet, VL: visible light.

Figure 1 clearly shows the change in appearance of the prepared samples. In the case of Ti coatings on bamboo, the color changes from slight metallic color (Ti@b) to light blue (Ti-cHT), which is similar to those cases of Al_2_O_3_ balls^29,30^. While for TiC-Ti coatings, the color changes from black (20TiC@b) to dark grey (20TiC-cHT), which is also similar to our previous works for Al_2_O_3_ balls^30,34^.

**Figure 2:**
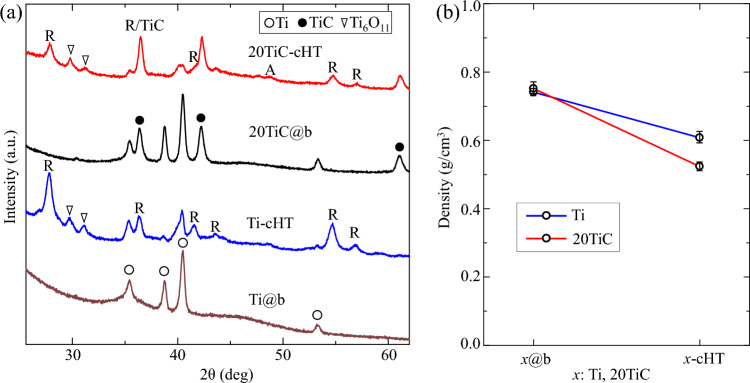
XRD patterns (**a**) and (**b**) density of the samples. A is anatase, and R is rutile.

XRD patterns and the density of the coatings on the bamboo, and their changes during the heat treatment in carbon powder are shown in Fig. 2. The diffraction peaks of the Ti@b sample reveal that Ti coatings (JCPDS # 44–1294) form on the bamboo block, while the diffraction peaks of the 20TiC@b sample at 35.9°, 41.7°, and 60.5° can be correspond to the crystal planes of (111), (200), and (220) from TiC (JCPDS # 32–1383), meaning the formed coatings containing Ti and TiC^30,34^. Compared with TiC micro-powder, the TiC nano-powder is easier to coat on the surface of bamboo. After heat treatment, the diffraction peaks of the Ti-cTH and 20TiC-cHT samples at 27.8°, 36.5°, 41.5°, 43.6°, 54.7°, and 56.9° can be attributed to the planes of (110), (101), (111), (210), (211) and (220) from rutile TiO_2_, respectively. It means that rutile TiO_2_ is formed. Also, the diffraction peaks at 27.8°, 36.5°, 41.5°, 43.6°, 54.7°, and 56.9° could be detected, corresponding to the nonstoichiometric Ti_6_O_11_ formed in the low oxygen heat treatment^35–37^. Notably, the residual Ti could be detected from the Ti-cHT sample, forming a TiO_2_/Ti structure. However, the diffraction peak at 41.7° indicates that a certain amount of TiC remained after heat treatment, possibly forming a TiO_2_/TiC heterojunction photocatalyst^38–40^. In addition, Fig. 2b shows the density change of the samples. The density of the Ti or TiC-Ti coatings on bamboo is around 0.8 g/cm^3^, then the density decreases to around 0.6 g/cm^3^ or lower after heat treatment, which is due to the degradation of cellulose and significant changes in structure^41–43^. Low density of the samples ensures that the samples can float in water.

### Surface morphology

**Figure 3:**
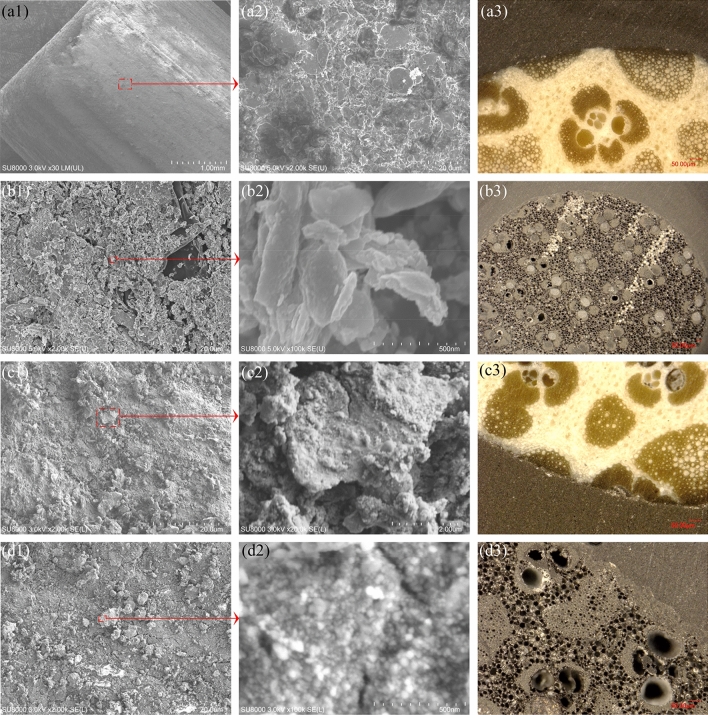
Comparison of surface morphology (1–3) and cross-Sect. (4) of the samples. (**a**) Ti@b, (**b**) Ti-cHT, (**c**) 20TiC@b, and (**d**) 20TiC-cTH.

Figure 3 presents the surface morphology and cross-section of the prepared samples. From Fig. 3a, it is obvious that the Ti coatings form on bamboo blocks, and the surface morphology of the Ti@b sample (Fig. 3a2) is similar to that of Ti coatings formed on Al_2_O_3_ balls^30,34^. Some naked parts (black area) could be found in Fig. 3a2, revealing the coatings are not completely continuous, which is more clear in Fig. 3b1. After heat treatment, the Ti-cHT sample exhibits the formed rutile TiO_2_ on Ti coatings, showing a block-like structure with a size ranging from 50 to 1200 nm. Compared with the change in cross-section during the heat treatment for Ti@b, Fig. 3b3 reveals that a lot of holes are formed due to the decomposition of cellulose^41–43^. While in the case of 20TiC@b as shown in Fig. 3c2, the bump-like structure forms on the Ti coatings could be attributed to the TiC powder, which is similar to our previous works^30,34^. Moreover, the cross-section of 20TiC@b (Fig. 3c3) is no significant change, compared to that of Ti@b (Fig. 3a3). After heat treatment for 20TiC@b, it evidently shows that the surface morphology is completely different from the Ti-cHT sample in size and quantity. Figure 3d2 demonstrates that the 20TiC-cHT sample possesses a nano-sized granular structures, size ranging from 20 to 50 nm. Notably, Fig. 3d3 shows that the cross-section of the 20TiC-cHT sample shows that more holes form during the heat treatment, compared to that of Ti-cHT (Fig. 3b3). It could be concluded that the noticeable difference in the surface morphology might be due to the coatings onto bamboo blocks and crystal structures formed in heat treatment. More importantly, the surface morphology and cross-section of the 20TiC-cHT sample indicate that it should possess an extremely highly accessible area.

**Figure 4:**
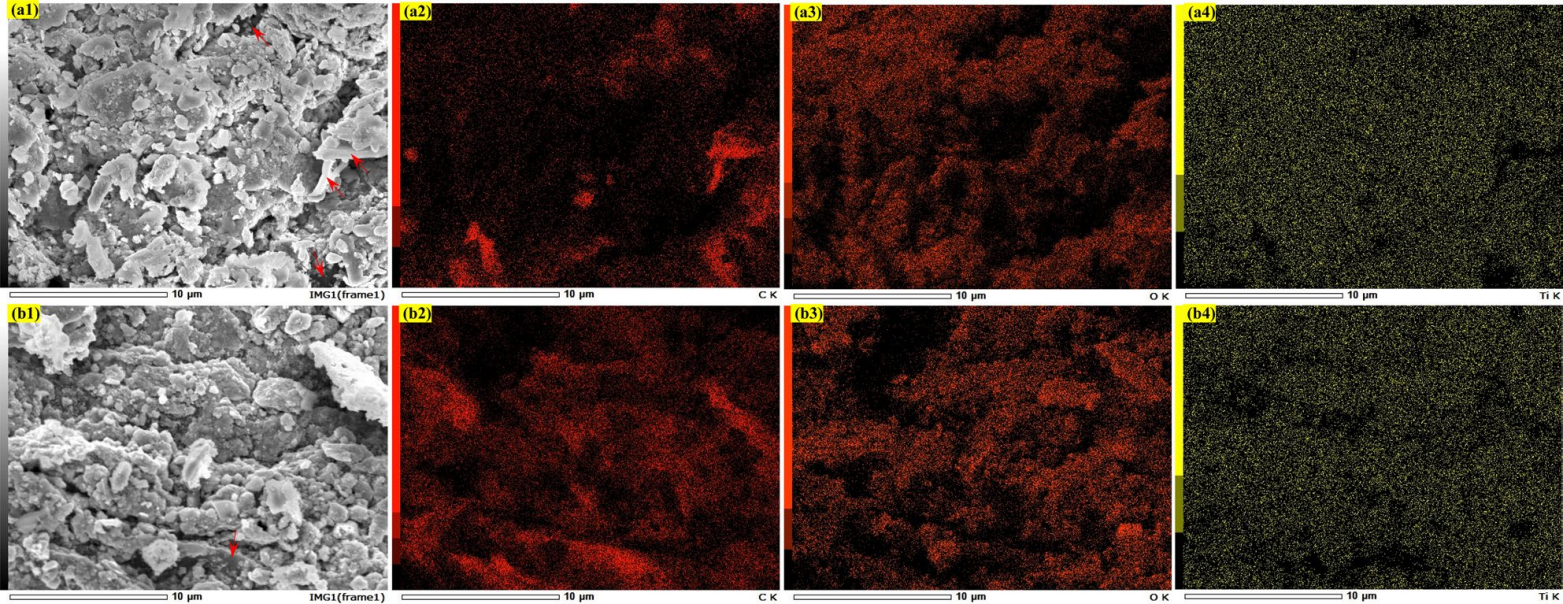
EDS analysis of the Ti-cHT and 20TiC-cHT samples. (**a**) Ti-cHT and (**b**) 20TiC-cHT.

The elemental distribution of the Ti-cHT and 20TiC-cHT samples was further evaluated based on EDS and mapping analyses, as shown in Fig. 4. The EDS analysis reveals the presence of considerable percentages of C, O, and Ti in the as-prepared coatings. From Fig. 4a, the distribution of Ti and C elements with colored dots in the mapping images confirms the formation of Ti coatings on the bamboo block. However, there is still some exposed parts (the areas pointed by the red arrows) of the bamboo, corresponding to the highlight in Fig. 4a2 and the dark in Fig. 4a4. In the selected area of the Ti-cHT sample, the distribution of Ti and O is relatively uniform, if C from bamboo and surface irregularity is taken into account. In other words, the surface of Ti can be considered to be oxidized into TiO_2_. While in the case of the 20TiC sample, comparing the surface morphology and Ti mapping, the area with the red arrow (Fig. 4b1) can be due to the uncoated parts of the bamboo. Meanwhile, further comparing the distribution of Ti and O, the highlighted area in C mapping can be attributed to the formed coatings, containing considerable percentages of TiC. Ignoring the influencing factors of C from bamboo and surface morphology, the distribution of Ti (from Fig. 4b4) is more uniform compared with that of Ti-cHT, owing to the coating material containing 20% TiC nano-powder. More importantly, considering the elemental distribution and surface morphology of the 20TiC sample, the considerable percentage of Ti could be concluded that the area corresponding to Ti is not completely oxidized into TiO_2_, which is also matched with the XRD results. In other words, a large area of TiC is not completely oxidized, thus forming a heterojunction of TiO_2_ and TiC, due to the stability of TiC.

### Visible light response and bonding environment

**Figure 5:**
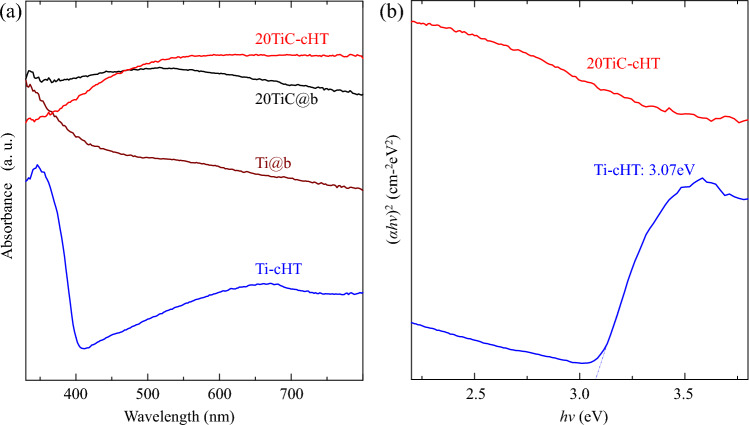
UV–vis absorption spectra of the prepared samples.

UV–vis spectroscopy is used to investigate the visible light response of the samples, as shown in Fig. 5. Compared with the light absorbance of TiO_2_ coatings in our previous works^29,30^, the absorbance edge of the Ti-cHT sample slightly moves towards the visible light region. Whereas the 20TiC-cHT sample exhibits light absorption in the almost visible light region, which can be owned to the formed nonstoichiometric TiO_2-x_ and the remaining TiC^29,44–46^.

**Figure 6:**
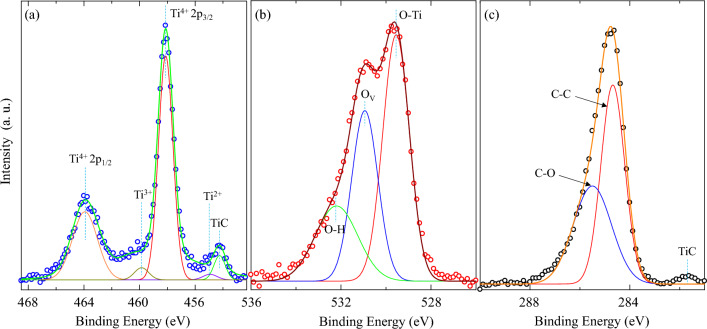
XPS spectra of the 20TiC-cHT sample. (**a**) Ti 2p, (**b**) O 1 s, and (**c**) C 1 s. Ov is oxygen vacancies.

XPS spectra of the 20TiC-cHT sample are shown in Fig. 6, while XPS results of the Ti-cHT sample could be referred to our previous works^39,47^. In Fig. 6a, the two peaks at 458.2 and 464.1 eV of binding energies correspond to Ti 2p_3/2_ and Ti 2p_1/2_, respectively, which is consistent with the published work^39,47^. While the peak at 454.3 eV could be attributed to the Ti-C bond from TiC^47–49^. Moreover, the slight peak at 459.9 eV corresponds to the formed Ti^3+^, neighboring to oxygen vacancies in rutile TiO_2_^47,50,51^. In the O 1 s spectra (Fig. 6b), the O 1 s peaks are asymmetric and yielded three chemical states of oxygen^47–51^. The peak at 529.5 eV can be attributed to the O^2-^ ions in the rutile TiO_2_^39,47^, while the peak at 530.9 eV can be owed to the formed oxygen vacancies during heat treatment to further cause the formation of nonstoichiometric TiO_2-x_, which is also matched with the formation of Ti^3+^^47,50,51^. What’s more, the related intensity of these two peaks indicates that a large amount of oxygen vacancies is formed. Furthermore, Fig. 6c shows that the peak at 281.7 eV could be due to the Ti-C bond from TiC, Meanwhile, the peak around 284.6 eV can be attributed to the formed C–C bond^47–49^.

### Photocatalytic activity and the related mechanism

**Figure 7:**
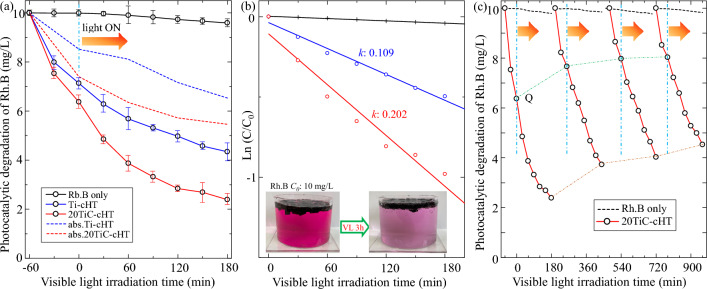
Comparison of the photocatalytic degradation and stability towards Rh.B solution under visible light irradiation. (**a**, **b**) Photocatalytic activity for the samples, (**c**) Cycle tests for the 20TiC-cHT sample. The color change of Rh.B is presented with the 20TiC-cHT sample.

The photodegradation of Rh.B solution under visible light irradiation presented in Fig. 7, it can be found that the photocatalytic activity of the 20TiC-cHT sample is higher than that of the Ti-cHT sample. The black line marked with Rh.B only in Fig. 7a indicates the stability of Rh.B solution under visible light irradiation. From Fig. 7a, it shows that the Rh.B solution concentration decreases by about 76% for the 20TiC-cHT sample, and about 56% for the Ti-cHT sample, respectively. The results clearly demonstrate that the two samples have excellent adsorption performance and photocatalytic activity, considering the quick change in concentration of Rh.B solution. Figure 7b clearly shows that the decomposition rate of the 20TiC-cHT sample is about 2 times higher than that of the Ti-cHT sample. Furthermore, the adsorption performance of related materials prepared from bamboo has also attracted much attention^10,52,53^. Therefore, the adsorption evalution was also performed in the dark, accompanying the photodegradation of Rh.B solution under visible light irradiation, as shown in dashed line in Fig. 7a. As expected, the Rh.B solution concentration also decreases by about 45% in the dark using the 20TiC-cHT sample (red dashed line) due to the remarkable adsorption, while about 35% for the Ti-cHT sample (blue dashed line). The details of Q and R are presented in Table 1.

**Table 1:** Comparison of the samples. Q_1h_ and Q_total_ are the samples with Rh.B in dark for 1 h and 4 h, respectively. R_3h_ is the sample with Rh.B under visible light. Q_1h_ + R_3h_ is the sample with Rh.B for 1 h in dark followed 3 h under visible light.

Sample	Q_1h_ (%)	Q_total_ (%)	R_3h_ (%)	Q_1h_ + R_3h_ (%)
Bamboo-cHT	21.5	35.3	21.1	38.1
Ti-cHT	30.6	35.7	48.4	56.6
20TiC-cHT	36.0	45.4	62.4	76.1

The stability of photocatalysis is a very important indicator from the application perspective, so the cycle tests of the 20TiC-cHT sample were carried out with a fresh Rh.B solution for each cycle. From Fig. 7c, it can be seen that the concentration of Rh.B solution in each cycle still decreases significantly and tends to be stable after 2 cycles, revealing satisfactory photocatalytic stability. Notably, the Q within 1 h for one cycle decreases from 36.0% to around 20% and the R keeps around 50%.

**Figure 8:**
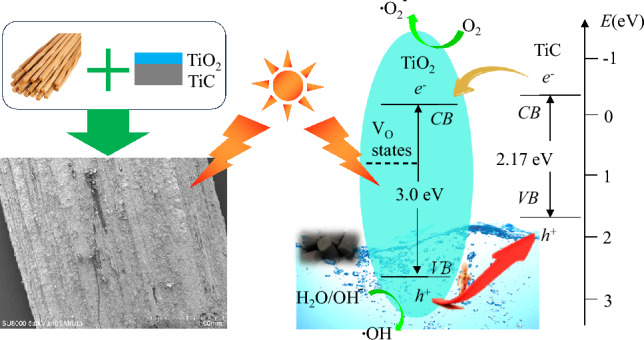
Schematic diagram of the improved Rh. B degradation efficiency. V_O_ represents the energy position formed by oxygen vacancies in the band gap.

The remarkable and stable photocatalytic performance of the TiO_2_/TiC@b fPC mainly originate from the more efficient visible light response (Fig. 5) due to the floatable property, which is due to the enhanced reaction at the water/air interface with the reduced light loss in water, and the possibly rapid delivery between oxygen and capture holes^26^. In addition to the efficient visible light response, the increased accessible area owing to the formed nano-sized granular structures (Fig. 3), and the possibly formed heterojunction of TiO_2_ and TiC (Figs. 2 and 6) also further enhance the photocatalytic performance. A large number of studies have shown that heterojunction photocatalysts can effectively enhance the charge transfer^54–56^. Considering the DBCs waste and the TiO_2_/TiC@b fPC, the probable mechanism for the photocatalytic decomposition of RhB dye has been proposed, as shown in Fig. 8. Under visible light irradiation, the TiO_2_/TiC can be excited to yield electrons and holes. The photoexcited holes in the valence band of TiO_2_ that can react with water to produce OH^**.**^radicals or transfer to the valence band of TiC^54^. Accompanied by the transferred holes from the valence band of TiO_2_, it will also generate electrons and holes in TiC^22,55^. Simultaneously, the photogenerated electrons will inevitably move to the conduction band, where the electrons can react with oxygen to form reactive O^2−^ radicals, to further decompose organic pollutants^54^. All these formed reactive radicals will enhance the photocatalytic decomposition of the Rh.B dye.

## Conclusions

The study indicates that DBCs waste has the potential to be used as fPC for water purification. The fPC of TiO_2_/TiC@b has been fabricated from TiC-Ti powders and DBCs by a specific coating method followed heat treatment in carbon powder, and the fPC exhibites excellent photocatalytic activity under visible light irradiation. TiO_2_ with rutile phase forms on the surface of TiC during the heat treatment, achieving a low-density of approximately 0.5233 g/cm^3^. The formed TiO_2_/TiC coatings on the bamboo are firm and uniform, having a particle size of 20–50 nm. A large amount of oxygen vacancies is generated that further favore narrowing the band gap of rutile TiO_2_. The heterojunction of TiO_2_ and TiC effectively inhibits the photogenerated electron–hole recombination via the charge transfer effect. Notably, the evaluation of Rh.B dye exhibits that the degradation efficiency of Rh.B is 62.4% within 3 h, while the dark adsorption observed to be 36.0% for 1 h. The excellent adsorption and photocatalytic performance of TiO_2_/TiC@b fPC are because of the enhanced reaction at the water/air interface due to the reduced light loss in water, improved visible-light response, increased accessible area, and charge transfer effect. Our proposed strategy of fabricating fPC onto the used DBCs waste is a simple, green feasible, low-cost, and mass-producible approach, and it provides some new opportunities in waste recycling and water treatment.

## Data Availability

The data that support the findings of this study are available from the article, or from the corresponding authors upon reasonable request.
